# Determinants of Cardiovascular Events After Simultaneous Pancreas Kidney Transplantation: A Retrospective Cohort Study

**DOI:** 10.7759/cureus.110399

**Published:** 2026-06-07

**Authors:** Steve Khalil, Zahidul Mondal, Neeraj Singh, Nooruddin S Hashmi, Jing Shen, Ronald P Pelletier, Mojgan Jalalzadeh

**Affiliations:** 1 Internal Medicine and Nephrology, Rutgers Robert Wood Johnson Medical School, New Brunswick, USA; 2 Internal Medicine, Nephrology and Transplant, Rutgers Robert Wood Johnson Medical School, New Brunswick, USA; 3 Medicine, Division of Gastroenterology, Rutgers Robert Wood Johnson Medical School, New Brunswick, USA; 4 Surgery, Robert Wood Johnson Barnabas Health, New Brunswick, USA

**Keywords:** cardiovascular risk, cold ischemic time, egfr, graft function, major adverse cardiovascular events, simultaneous pancreas–kidney transplantation, triglyceride–glucose index

## Abstract

Background

Simultaneous pancreas-kidney transplantation (SPK) provides important metabolic and renal benefits, but cardiovascular complications remain a source of post-transplant morbidity and mortality. Factors associated with major adverse cardiovascular events (MACE) in contemporary SPK populations remain incompletely characterized.

Methods

We performed a single-center retrospective cohort analysis of 80 adult SPK recipients transplanted between 2018 and 2025. The primary endpoint was post-transplant MACE, defined as a composite of nonfatal myocardial infarction, nonfatal stroke, cardiovascular death, heart failure hospitalization, or clinically significant arrhythmia. MACE was evaluated as a binary outcome. The multivariable logistic regression model included one-month estimated glomerular filtration rate (eGFR) and kidney cold ischemic time.

Results

MACE occurred in 14 recipients (17.5%). Compared with recipients without MACE, those with MACE had more frequent post-transplant insulin use (P=0.013), longer kidney cold ischemic time (P=0.015), and higher donor body mass index (P=0.001). The triglyceride-glucose index was not significantly associated with MACE. In multivariable analysis, higher one-month eGFR was associated with lower odds of MACE (adjusted odds ratio, 0.61 per 10 mL/min/1.73 m² increase; 95% confidence interval (CI), 0.38-0.97; P=0.040). Kidney cold ischemic time showed a borderline association with MACE (adjusted odds ratio per hour, 1.28; 95% CI, 1.00-1.67; P=0.057). Model discrimination was modest, with an area under the curve of 0.68.

Conclusions

Among SPK transplant recipients, lower early kidney allograft function was associated with higher observed odds of post-transplant cardiovascular events. The triglyceride-glucose (TyG) index was not independently associated with MACE. Because few events occurred, these findings should be interpreted as exploratory and validated in larger studies.

## Introduction

Simultaneous pancreas-kidney transplantation (SPK) is an established therapeutic option for selected patients with insulin-dependent diabetes mellitus and end-stage kidney disease. By restoring renal function and endogenous insulin secretion, SPK provides durable renal replacement, improves glycemic control, reduces the need for exogenous insulin, and may favorably affect lipid and blood pressure parameters [1-3]. These metabolic and renal benefits may improve quality of life and survival compared with the remaining dialysis-dependent patients and may offer advantages over kidney-alone transplantation in appropriately selected patients. Prior work has also shown that SPK transplantation can substantially reduce predicted cardiovascular risk, with both kidney and pancreas graft function contributing to this benefit [4].

Despite the clinical benefits of SPK transplantation, cardiovascular complications continue to represent an important source of post-transplant morbidity and mortality. Many SPK recipients enter transplantation with long-standing diabetes, advanced kidney disease, and pre-existing macrovascular injury, all of which may contribute to persistent cardiovascular vulnerability after surgery [5-8]. Therefore, major adverse cardiovascular events (MACE), including myocardial infarction, stroke, and cardiovascular death, may still occur despite improved glycemic control. In a UK registry study of 1,699 SPK recipients, 7.8% developed MACE within five years, and older age, prior myocardial infarction, prior stroke, and donor hypertension were identified as significant predictors [6]. These findings suggest that glycemic normalization after SPK does not fully eliminate established atherosclerotic disease or reverse the effects of long-standing cardiometabolic injury [4-6]. Cardiovascular risk may also be concentrated early after transplantation, supporting the need for timely post-transplant risk assessment and closer surveillance. Interpretation of the existing literature remains challenging because prior studies have used different MACE definitions, follow-up periods, and cohort sizes [2,3,6,7].

Although traditional metabolic risk factors contribute to cardiovascular disease burden, post-transplant outcomes may also be influenced by factors specific to transplantation, including early graft performance, ischemia-reperfusion injury, and perioperative stress [8,9]. Renal allograft function is particularly relevant because impaired kidney function may perpetuate chronic kidney disease-related mechanisms, such as inflammation, endothelial dysfunction, and accelerated vascular disease even after transplantation [4,8]. Consistent with this relationship, the Folic Acid for Vascular Outcome Reduction in Transplantation (FAVORIT) trial demonstrated that for kidney transplant recipients whose estimated glomerular filtration rate (eGFR) values are below 45 mL/min/1.73 m², each 5 mL/min/1.73 m² higher eGFR was associated with a 15% lower risk of cardiovascular disease and death [8].

Concurrently, metabolic risk assessment has expanded beyond traditional markers. The triglyceride-glucose (TyG) index has been used as a practical surrogate of insulin resistance and may provide additional information about cardiovascular vulnerability beyond standard clinical risk factors [9-11]. However, in SPK recipients, the extent to which metabolic indices versus transplant-related variables contribute to cardiovascular events remains uncertain, particularly during the early post-transplant period.

This study was designed to clarify the burden and correlates of post-transplant cardiovascular events after SPK transplantation. Specifically, the objectives were: (1) to describe the incidence of post-transplant major adverse cardiovascular events (MACE) after SPK transplantation; (2) to examine whether metabolic markers, including glycated hemoglobin (HbA1c), lipid parameters, and the TyG index, were associated with post-transplant MACE; and (3) to explore whether transplant-related factors, including early kidney allograft function and cold ischemic time, were associated with post-transplant MACE. We hypothesized that post-transplant cardiovascular events would be more closely associated with early graft function and transplant-related factors than with baseline metabolic markers alone.

## Materials and methods

Study design and population

This single-center retrospective cohort study evaluated adult patients who underwent SPK at an academic medical center from January 2018 through December 2025. The institutional review board approved the study protocol (IRB Pro2025002041) and waived informed consent from the patients.

Recipients were included if they were at least 18 years old at the time of transplantation and had sufficient post-transplant clinical follow-up to assess cardiovascular outcomes. Recipients were excluded if data on the primary outcome, MACE, were unavailable or if follow-up documentation was inadequate to ascertain whether a cardiovascular event had occurred.

Data sources and variables

Recipient-level demographic and clinical information was obtained from institutional transplant databases and electronic health records. Baseline variables included age at transplantation, sex, race/ethnicity, body mass index (BMI), diabetes type, and smoking status. Pre-transplant renal replacement therapy (RRT) duration was defined as the interval from RRT initiation to transplantation. Calculated panel-reactive antibody (CPRA) percentage was also collected, as defined by the Organ Procurement and Transplantation Network/United Network for Organ Sharing (OPTN/UNOS) allocation methodology [11]. Metabolic data included glycated hemoglobin (HbA1c), fasting plasma glucose, and fasting lipid values, including total cholesterol, low-density lipoprotein cholesterol, high-density lipoprotein cholesterol, and triglycerides. The TyG index was calculated using the established formula [12]:

$\ln\left(\frac{\text{fasting triglycerides (mg/dL)} \times \text{fasting glucose (mg/dL)}}{2}\right)$.

Because this index is inherently logarithmic, no further transformation was performed. Additional laboratory values, including amylase, lipase, and C-peptide, were recorded when available.

Pre-transplant comorbidities included hypertension and prior macrovascular disease. Macrovascular disease was defined as a documented history of myocardial infarction, percutaneous coronary intervention, congestive heart failure, or cerebrovascular accident. Post-transplant cardiovascular medication use, including statin and antihypertensive therapy, was abstracted from the medical record. Because pre-transplant medication documentation was incomplete and inconsistent, these variables were not analyzed.

Donor and transplant-related data included donor type, donor age, sex, BMI, race/ethnicity, kidney cold ischemic time, pancreas preservation time, and Kidney Donor Profile Index (KDPI), as derived from the Kidney Donor Risk Index and described by OPTN methodology [13]. Donors were classified as donation after brain death (DBD) or donation after circulatory death (DCD).

Post-transplant variables included delayed graft function (DGF), serial serum creatinine values, eGFR calculated using the Chronic Kidney Disease Epidemiology Collaboration (CKD-EPI) equation [14] at one, 12, and 36 months, and insulin use after transplantation. Delayed graft function was defined as the need for dialysis within the first post-transplant week.

Outcome definitions

The primary endpoint was the occurrence of post-transplant MACE during follow-up. In this study, MACE was defined as a composite outcome that included nonfatal myocardial infarction, nonfatal stroke, cardiovascular death, hospitalization for heart failure, or clinically significant arrhythmia. Clinically significant arrhythmia was defined as a documented arrhythmia requiring medical evaluation, treatment, hospitalization, cardiology consultation, antiarrhythmic therapy, anticoagulation initiation for atrial arrhythmia, or device-based intervention. This definition is broader than the conventional three-point MACE definition, which typically includes cardiovascular death, nonfatal myocardial infarction, and nonfatal stroke [15]. We selected this expanded definition to capture the clinically important cardiovascular complications relevant to the SPK transplant population, including heart failure hospitalization and arrhythmia, which may occur in the perioperative and post-transplant setting. Because this expanded definition differs from the conventional three-point MACE, comparisons with prior studies using narrower MACE definitions should be interpreted with caution.

The follow-up began on the date of transplantation and continued until December 30, 2025, death, or last available clinical follow-up, whichever occurred first. Cardiovascular events were identified by a review of available clinical documentation, including transplant follow-up notes, cardiology notes, hospital discharge summaries, emergency department records, diagnostic testing, and death documentation when available. Events were not independently adjudicated by a blinded committee; therefore, outcome classification was based on documentation in the medical record.

Because precise dates for cardiovascular events were not consistently available, MACE was evaluated as a binary yes/no outcome rather than as a time-to-event outcome. As a result, the analysis did not assess event timing, censoring, or changes in cardiovascular risk over time.

Statistical analysis

Descriptive statistics were used to summarize the recipient, donor, and transplant characteristics. Continuous variables are presented as means with standard deviations (SD) when normally distributed or medians with interquartile ranges (IQR) when skewed; categorical variables are reported as counts and percentages.

Comparisons between recipients with and without post-transplant MACE were performed using chi-square or Fisher's exact tests for categorical variables and Student's t-test for normally distributed continuous variables, or the Wilcoxon rank-sum test for non-normally distributed continuous variables, as appropriate based on data distribution. Fisher’s exact test was selected for sparse contingency tables, particularly when expected cell counts were less than five.

To evaluate the factors associated with post-transplant MACE, multivariable logistic regression was performed. Because only 14 MACE occurred, the multivariable model was intentionally limited to reduce the risk of overfitting, consistent with events-per-variable and prediction-model sample-size considerations [16,17]. With two covariates and 14 outcome events, the events-per-variable ratio was 7, which is below the commonly cited threshold of 10. Therefore, regression estimates, odds ratios, confidence intervals, and P values should be interpreted cautiously. Candidate variables were selected a priori based on clinical relevance and prior literature, and no automated or stepwise variable selection procedures were used. The final adjusted model included one-month eGFR and kidney cold ischemic time.

Because one-month eGFR was measured after transplantation and exact cardiovascular event dates were not consistently available, one-month eGFR was treated as an intermediate marker of early graft function rather than as a baseline predictor. Therefore, associations involving one-month eGFR were interpreted as associative rather than causal or predictive.

Continuous variables were handled consistently across analyses. Distributional properties were assessed using visual inspection and summary statistics. Variables with approximately normal distributions were analyzed on their original scale, whereas skewed variables were summarized using medians and interquartile ranges for descriptive analyses.

For regression modeling, continuous variables were retained on their original scales. They were expressed using clinically meaningful units, including per 10 mL/min/1.73 m² increase in eGFR, per hour increase in cold ischemic time, and per 10 mg/dL increment for lipid parameters. The TyG index, which is inherently log-transformed by definition, was included as a continuous variable without additional transformation.

Adjusted odds ratios (ORs) with 95% confidence intervals (CIs) were reported. Given the small number of outcome events, model estimates were interpreted cautiously, as low event rates can lead to instability and imprecision in effect estimates.

Model discrimination was assessed using the area under the receiver operating characteristic curve (AUC), and calibration was evaluated using the Hosmer-Lemeshow goodness-of-fit test. Given the limited number of outcome events, findings were considered hypothesis-generating.

Variables with missing data were analyzed using available-case analysis. Denominators were reported in tables where data were incomplete, particularly for metabolic and longitudinal laboratory variables.

All statistical tests were two-sided, and P values <0.05 were considered statistically significant. Analyses were conducted using R version 4.4.1 (R Foundation for Statistical Computing, Vienna, Austria).

## Results

Study characteristics

A total of 80 adult SPK transplant recipients were included in the study. During follow-up, 14 recipients (17.5%) experienced post-transplant MACE. Baseline demographic characteristics were generally comparable between recipients with and without MACE (Table 1). No statistically significant differences were observed in age at transplantation, sex distribution, BMI, diabetes type, smoking status, or race/ethnicity between groups.

**Table 1:** Baseline recipient and donor characteristics stratified by post-transplant major adverse cardiovascular event (MACE) status Continuous variables are summarized as mean±standard deviation (SD) or median with interquartile range, depending on distribution. Categorical variables are shown as n (%) when complete and as n/N (%) when denominators vary because of missing data. Between-group comparisons used Student’s t-test or the Wilcoxon rank-sum test for continuous variables and the chi-square test or Fisher’s exact test for categorical variables, as appropriate. Fisher’s exact test was used for sparse contingency tables or when expected cell counts were less than 5. Denominators may vary across variables because of missing data and are provided where applicable. Abbreviations: BMI, body mass index; CPRA, calculated panel-reactive antibody; DBD, donation after brain death; DCD, donation after circulatory death; KDPI, Kidney Donor Profile Index; MACE, major adverse cardiovascular events; SD, standard deviation; IQR, interquartile range.

Variable	No MACE (n=66)	MACE (n=14)	Statistic	P value
Recipient characteristics				
Age at transplant, mean±SD (years)	46.7±10.4	47.2±9.9	t=0.18	0.858
Male sex, n (%)	51 (77.3)	13 (92.9)	Fisher	0.280
BMI, mean±SD (kg/m²)	25.9±3.7	27.5±4.2	t=1.28	0.217
Race/ethnicity, n (%)			Fisher	0.847
• Asian, non-Hispanic	14 (21.2)	3 (21.4)		
• Black, non-Hispanic	17 (25.8)	2 (14.3)		
• Hispanic/Latino	19 (28.8)	5 (35.7)		
• White, non-Hispanic	16 (24.2)	4 (28.6)		
Smoking, any, n (%)	27 (39.1)	7 (63.6)	Fisher	0.189
Smoking status, n (%)			Fisher	0.404
Never	39 (59.1)	6 (42.9)		
Former	19 (28.8)	5 (35.7)		
Current	7 (10.6)	3 (21.4)		
Missing	1 (1.5)	0 (0)		
Diabetes type, n (%) Type 1, Type 2	26 (39.4), 40 (60.6)	5 (35.7), 9 (64.3)	Fisher	1.00
Years on dialysis prior to transplant, mean±SD	1.86±1.86	1.86±0.84	t=0.01	0.999
Pre-transplant renal replacement duration, median (IQR), years	1.4 (0.9-2.2)	1.8 (1.2-2.8)	W=364.0	0.217
Prior macrovascular comorbidity, n (%)	32 (48.5)	3 (21.4)	Fisher	0.080
CPRA, mean±SD (%)	13.2±25.6	9.2±11.9	t=0.79	0.436
Post-transplant follow-up duration, mean±SD (years)	2.36±1.90	1.74±1.80	t=1.15	0.262
Donor characteristics			Fisher	0.690
Donor type DBD, n (%)	56 (84.8)	11 (78.6)		
Donor type DCD, n (%)	10 (15.2)	3 (21.4)		
Donor age, mean±SD (years)	30.1±11.2	32.9±11.9	t=0.81	0.426
Donor male sex, n (%)	38 (57.6)	7 (50.0)	Fisher	0.768
Donor BMI, mean±SD (kg/m²)	21.1±11.3	27.5±4.6	t=3.44	0.001
Kidney cold ischemic time, mean±SD (hours)	13.6±2.7	15.6±2.5	t=2.64	0.015
Pancreas preservation time, mean±SD (hours)	11.6±2.4	12.9±2.1	t=1.86	0.079
KDPI, mean±SD (%)	18.2±13.7	14.7±12.0	t=0.97	0.343

Some metabolic parameters were numerically higher among recipients who developed MACE, although these differences were not statistically significant. Mean HbA1c was higher in the MACE group than in recipients without MACE (8.19±1.94% vs 7.42±1.60%; P=0.200), and fasting glucose was also numerically higher (127±71 vs 109±42.6 mg/dL; P=0.391) (Table 2). Lipid parameters were not significantly different between groups, including triglycerides (318±507 vs 134±66.5 mg/dL; P=0.310), total cholesterol (P=0.192), low-density lipoprotein (LDL) cholesterol (P=0.499), and high-density lipoprotein (HDL) cholesterol (P=0.541). The TyG index, analyzed as a surrogate marker of insulin resistance, was not significantly associated with MACE (8.90±0.80 vs 8.69±0.57; P=0.507) (Table 2).

**Table 2:** Post-transplant clinical, metabolic, and treatment characteristics stratified by post-transplant MACE status Continuous variables are summarized as mean±standard deviation or median with interquartile range, depending on distribution. Categorical variables are shown as n (%) when complete and as n/N (%) when denominators vary because of missing data. Between-group comparisons used Student’s t-test or the Wilcoxon rank-sum test for continuous variables and the chi-square test or Fisher’s exact test for categorical variables, as appropriate. Fisher’s exact test was used for sparse contingency tables or when expected cell counts were less than 5. Denominators may vary across variables because of missing data and are provided where applicable. Abbreviations: eGFR, estimated glomerular filtration rate; HbA1c, glycated hemoglobin; HDL, high-density lipoprotein; IQR, interquartile range; LDL, low-density lipoprotein; MACE, major adverse cardiovascular events; SD, standard deviation; TyG, triglyceride-glucose.

Variable	No MACE (n=66)	MACE (n=14)	Statistic	P value	
Graft status					
Delayed graft function, n (%)	19 (28.8)	2 (14.3)	Fisher	0.329	
Serum creatinine at 1 month, mean±SD (mg/dL)	1.58 ± 0.72	1.83 ± 0.56	t=1.41	0.173	
eGFR at 1 month, mean±SD (mL/min/1.73 m²)	48.9 ± 13.4	40.6 ± 15.1	t=1.84	0.085	
Serum creatinine at 12 months, mean±SD (mg/dL)	1.28 ± 0.39	2.24 ± 1.40	t=1.80	0.121	
eGFR at 12 months, mean±SD (mL/min/1.73 m²)	53.3 ± 11.0	39.2 ± 19.0	t=2.15	0.060	
Serum creatinine at 36 months, mean±SD (mg/dL)	1.98 ± 1.88	2.52 ± 1.93	t=0.53	0.626	
eGFR at 36 months, mean±SD (mL/min/1.73 m²)	47.2 ± 15.9	41.5 ± 20.2	t=0.54	0.621	
Post-transplant insulin use, n (%)	5 (7.6)	5 (35.7)	Fisher	0.013	
Metabolic parameters					
HbA1c mean±SD (%), available n: No MACE/MACE=56/13	7.42 ± 1.60	8.19 ± 1.94	t=1.34	0.200	
Fasting glucose, mean±SD (mg/dL), available n: No MACE/MACE=65/13	109 ± 42.6	127 ± 71.0	t=0.89	0.391	
Total cholesterol, mean ± SD (mg/dL), available n: No MACE/MACE = 50/9	138 ± 38.8	171 ± 66.7	t=1.41	0.192	
LDL, mean±SD (mg/dL), available n: No MACE/MACE = 49/9	71.8 ± 31.4	80.4 ± 34.7	t=0.70	0.499	
HDL, mean±SD (mg/dL), available n: No MACE/MACE = 50/9	49.5 ± 16.8	45.4 ± 17.7	t=0.63	0.541	
Triglycerides, mean±SD (mg/dL), available n: No MACE/MACE = 50/9	134 ± 66.5	318 ± 507	t=1.08	0.310	
Natural logarithm triglyceride-glucose (TyG) index, mean±SD, available n: No MACE/MACE=49/8	8.69 ± 0.57	8.90 ± 0.80	t=0.69	0.507	
Amylase, median (IQR), available n: No MACE/MACE=65/12	96 (70–114)	62.5 (46.8–133.8)	W=462.5	0.312	
Lipase, median (IQR), available n: No MACE/MACE=65/12	48 (31–75)	26.5 (16.5–77.3)	W=477.5	0.222	
C-peptide, median (IQR), available n: No MACE/MACE=50/9	5.4 (3.9–7.5)	4.3 (2.4–5.7)	W=288	0.188	
Post-transplant medication use					
Statin use, n (%)	45 (68.2)	11 (78.6)	Fisher	0.536	
Antihypertensive use, n (%)	46 (69.7)	9 (64.3)	Fisher	0.755	

Pre-transplant macrovascular comorbidities, including myocardial infarction, percutaneous coronary intervention, congestive heart failure, and cerebrovascular accident, were present in 48.5% of recipients without MACE compared with 21.4% of recipients with MACE (P=0.080). Post-transplant use of statins and antihypertensive medications was similar between the groups.

Donor and transplant characteristics

Donor and transplant characteristics were generally comparable between recipients with and without MACE (Table 1), including donor age, sex, KDPI, calculated panel-reactive antibody (CPRA), and donor type. Donor BMI was significantly higher among recipients who developed MACE than among those without MACE (27.5±4.6 vs 21.1±11.3 kg/m²; P=0.001). Ischemic preservation times were also longer in the MACE group. Kidney cold ischemic time was significantly longer among recipients who developed MACE (15.6±2.5 vs 13.6±2.7 hours; P=0.015), and pancreas preservation time was numerically longer but did not reach statistical significance (12.9±2.1 vs 11.6±2.4 hours; P=0.079).

Post-transplant cardiovascular events

**Figure 1:**
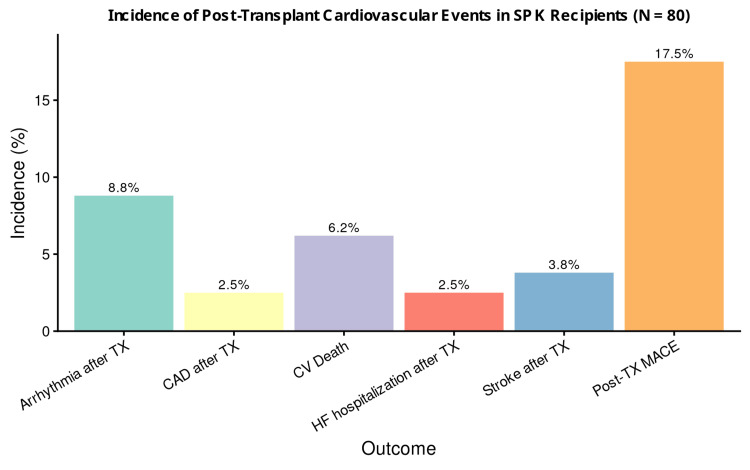
Cardiovascular events after simultaneous pancreas-kidney transplantation. Bar chart showing the percentage of recipients who experienced each cardiovascular event during follow-up. Major adverse cardiovascular events (MACE) was defined as a composite of nonfatal myocardial infarction, nonfatal stroke, cardiovascular death, heart failure hospitalization, or clinically significant arrhythmia. Percentages were calculated using the full study cohort as the denominator (n=80). Event categories are not mutually exclusive.

During follow-up, 14 recipients (17.5%) experienced post-transplant MACE (Table 3). Myocardial infarction occurred in two recipients (2.5%), stroke in three recipients (3.8%), and cardiovascular death in five recipients (6.2%). Additional cardiovascular events included heart failure hospitalization in two recipients (2.5%) and arrhythmia in seven recipients (8.8%). The distribution of cardiovascular events is shown in Figure 1.

**Table 3:** Incidence of cardiovascular events following simultaneous pancreas-kidney transplantation Values are shown as n (%) using the full study cohort as the denominator (n=80). Major adverse cardiovascular events (MACE) was defined as a composite of nonfatal myocardial infarction, nonfatal stroke, cardiovascular death, heart failure hospitalization, or clinically significant arrhythmia. Individual event categories are listed separately and are not mutually exclusive.

Outcome	Number of recipients, n (%)
Myocardial infarction	2 (2.5)
Stroke	3 (3.8)
Cardiovascular death	5 (6.2)
Heart failure hospitalization	2 (2.5)
Arrhythmia	7 (8.8)
Major adverse cardiovascular events (MACE)	14 (17.5)

Post-transplant renal function and metabolic outcomes

Post-transplant renal function showed partial differences between recipients with and without MACE (Table 2). Delayed graft function occurred at similar frequencies in the two groups (28.8% vs 14.3%; P=0.329). At one month after transplantation, recipients who developed MACE had lower eGFR than those without MACE (40.6±15.1 vs 48.9±13.4 mL/min/1.73 m²; P=0.085). At 12 months, the MACE group had numerically higher serum creatinine and lower eGFR, with the eGFR difference approaching statistical significance (39.2±19.0 vs 53.3±11.0 mL/min/1.73 m²; P=0.060). By 36 months, renal function measures were no longer significantly different between groups.

**Figure 2:**
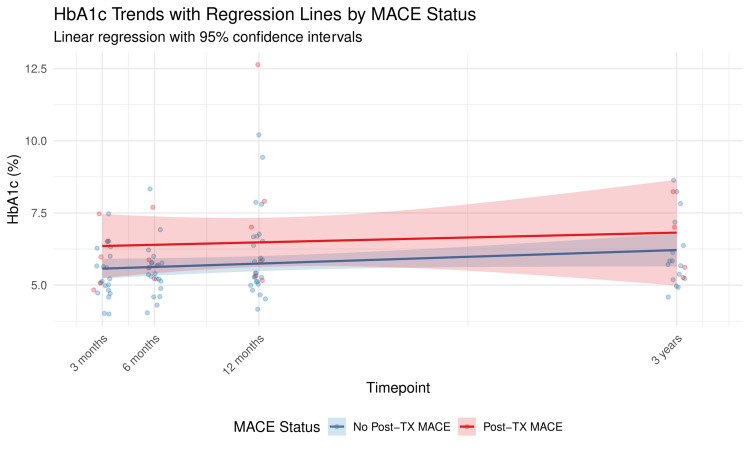
Longitudinal HbA1c values after simultaneous pancreas-kidney (SPK) transplantation, stratified by MACE status. Scatter plot showing HbA1c measurements over time in SPK transplant recipients grouped by post-transplant MACE status. Solid lines show fitted linear trends, and shaded bands represent 95% confidence intervals. Measurements are shown at three months, six months, 12 months, and three years after transplantation. MACE: Major adverse cardiovascular events.

Post-transplant insulin use was more frequent among recipients who developed MACE than among those without MACE (35.7% vs 7.6%; P=0.013) (Figure 2). This association should be interpreted cautiously, given the limited number of outcome events.

Univariable and multivariable logistic regression analysis

Univariable logistic regression results are presented in Table 4. Kidney cold ischemic time was significantly associated with higher odds of MACE (odds ratio (OR) 1.32 per hour; 95% confidence interval (CI), 1.05-1.66; P=0.019), whereas higher 12-month eGFR was associated with lower odds of MACE (OR 0.53 per 10 mL/min/1.73 m²; 95% CI, 0.32-0.87; P=0.011). One-month eGFR showed a borderline association with MACE (OR 0.67 per 10 mL/min/1.73 m²; 95% CI, 0.44-1.01; P=0.057). Other variables, including age, sex, smoking status, HbA1c, LDL cholesterol, insulin use, statin use, antihypertensive use, prior macrovascular disease, donor type, and the TyG index, were not significantly associated with MACE in univariable models.

**Table 4:** Univariable logistic regression analysis of factors associated with post-transplant major adverse cardiovascular events (MACE) Continuous variables are reported using the units shown in the table. Categorical variables are shown as n/N (%) where applicable. The TyG index is logarithmic by definition and was analyzed without additional transformation. Because the number of MACE cases was limited, odds ratio estimates may be imprecise and should be interpreted cautiously. Denominators may vary across variables because of missing data and are provided where applicable. Abbreviations: CHF, congestive heart failure; CI, confidence interval; CVA, cerebrovascular accident; DBD, donation after brain death; DCD, donation after circulatory death; eGFR, estimated glomerular filtration rate; HbA1c, glycated hemoglobin; LDL, low-density lipoprotein; MACE, major adverse cardiovascular events; MI, myocardial infarction; OR, odds ratio; SD, standard deviation; TyG, triglyceride-glucose.

Predictor	No MACE raw data	MACE raw data	OR (95% CI)	p-value
Age at transplant (per year), mean±SD	46.7±10.4	47.2±9.9	1.01 (0.95-1.06)	0.859
Male sex (vs female), n/N (%)	51/66 (77.3)	13/14 (92.9)	3.82 (0.46-31.66)	0.214
Smoking status (ref: never)	39 (59.1)	6 (42.9)	1.0 (ref)	
Former vs never	19 (28.8)	5 (35.7)	1.71 (0.46-6.32)	0.421
Current vs never	7 (10.6)	3 (21.4)	2.79 (0.56-13.83)	0.210
HbA₁c (per 1%), mean±SD	7.42±1.60	8.19±1.94	1.29 (0.92-1.82)	0.140
LDL (per 10 mg/dL), mean±SD	71.8±31.4	80.4±34.7	1.09 (0.88-1.35)	0.450
1-month eGFR (per 10 mL/min/1.73 m²), mean±SD	48.9±13.4	40.6±15.1	0.67 (0.44-1.01)	0.057
12-month eGFR (per 10 mL/min/1.73 m²), mean±SD	53.3±11.0	39.2±19.0	0.53 (0.32–0.87)	0.011
eGFR at 3 years (per 10 mL/min/1.73 m²), mean±SD	47.2±15.9	41.5±20.2	0.82 (0.46–1.47)	0.507
Insulin use (yes vs no), n/N (%)	5/66 (7.6)	5/14 (35.7)	2.78 (0.78–9.90)	0.115
Statin use (yes vs no), n/N (%)	45/66 (68.2)	11/14 (78.6)	1.71 (0.43–6.79)	0.445
Antihypertensive use (yes vs no), n/N (%)	46/66 (69.7)	9/14 (64.3)	0.78 (0.23–2.63)	0.692
Pre-transplant coronary artery disease, n/N (%)	20/66 (30.3%)	2/14 (14.3%)	0.38 (0.08–1.83)	0.226
Pre-transplant CHF, n/N (%)	11/66 (16.7%)	1/14 (7.1%)	0.38 (0.05–3.25)	0.380
Pre-transplant MI/CHF/CVA, n/N (%)	2/66 (3.0%)	0/14 (0%)	0.29 (0.07–1.13)	0.075
Donor type (DCD vs DBD), n/N (%)	10/66 (15.2)	3/14 (21.4)	1.53 (0.36–6.47)	0.565
Natural logarithm triglyceride–glucose (TyG) index, mean±SD	8.69±0.57	8.90±0.80	1.76 (0.51-6.14)	0.372
Kidney cold ischemic time (hours), mean±SD	13.6±2.7	15.6±2.5	1.32 (1.05–1.66)	0.019

**Figure 3:**
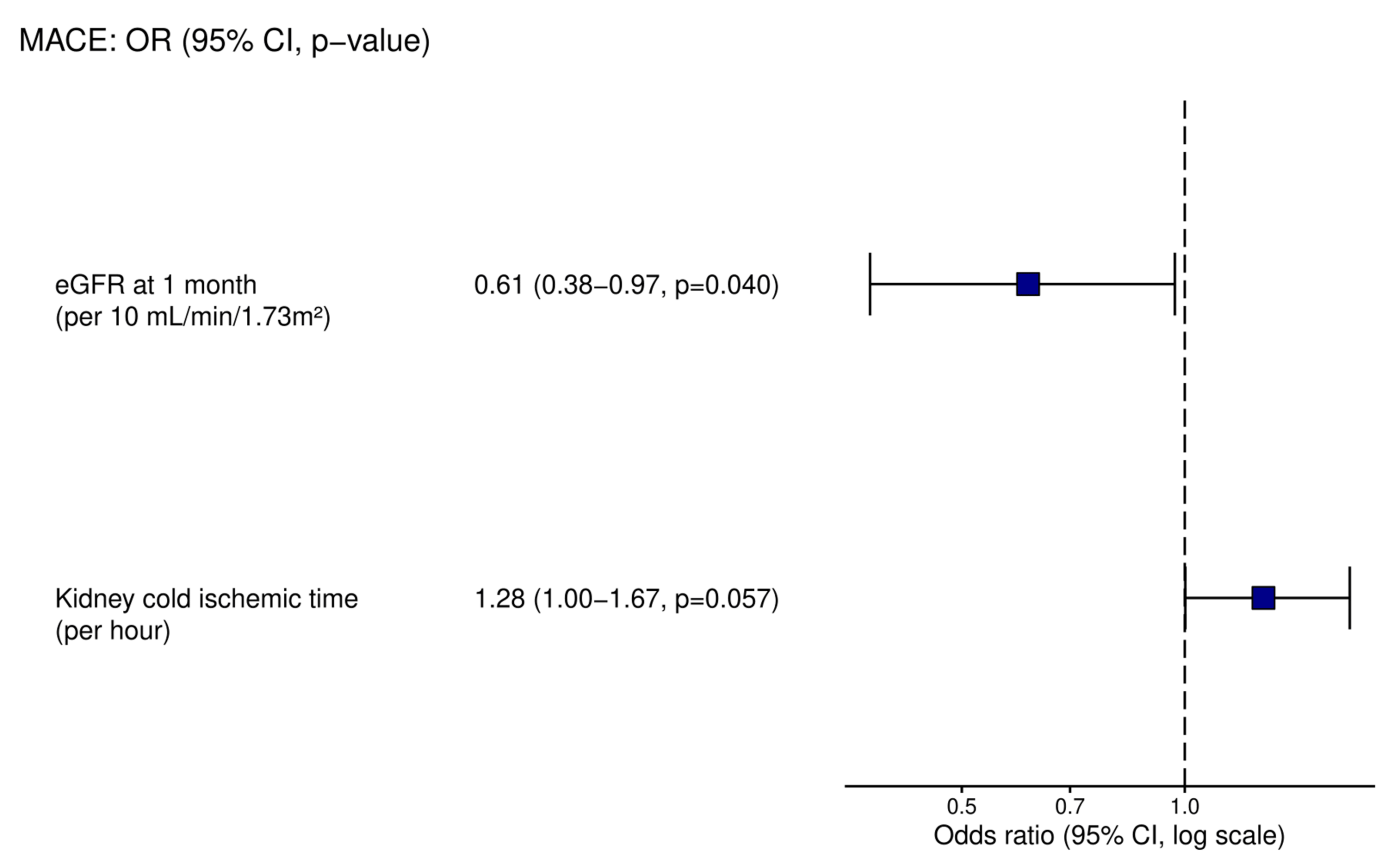
Adjusted associations with post-transplant major adverse cardiovascular events. Forest plot showing adjusted odds ratios and 95% confidence intervals for the two variables included in the final multivariable model: one-month estimated glomerulation filtration rate (eGFR) and kidney cold ischemic time. eGFR was modeled per 10 mL/min/1.73 m² increase, and kidney cold ischemic time was modeled per 1-hour increase. Estimates should be interpreted cautiously because of the limited number of outcome events.

To reduce the risk of overfitting, the multivariable model was limited to two clinically relevant predictors: one-month eGFR and kidney cold ischemic time (Table 5). In the adjusted model, higher one-month eGFR was associated with lower observed odds of MACE (adjusted OR, 0.61 per 10 mL/min/1.73 m² increase; 95% CI, 0.38-0.97; P=0.040). Kidney cold ischemic time showed a borderline association but did not reach statistical significance (adjusted OR per hour, 1.28; 95% CI, 1.00-1.67; P=0.057). The adjusted estimates are shown in Figure 3.

**Table 5:** Multivariable logistic regression analysis of factors associated with post-transplant MACE Odds ratios are reported with 95% confidence intervals. Continuous variables were modeled using the units shown in the table. Because only 14 MACE occurred, estimates may be unstable and should be interpreted cautiously. Abbreviations: CI, confidence interval; eGFR, estimated glomerular filtration rate; MACE, major adverse cardiovascular events; OR, odds ratio.

Predictor	No MACE raw data	MACE raw data	Adjusted OR (95% CI)	P value
1-month eGFR (per 10 mL/min/1.73 m² increase)	48.9±13.4	40.6±15.1	0.61 (0.38-0.97)	0.040
Kidney cold ischemic time (per hour)	13.6±2.7	15.6±2.5	1.28 (1.00-1.67)	0.057

Because only 14 MACE events occurred, the adjusted estimates from the multivariable model should be interpreted with caution. Confidence intervals were wide, reflecting the small number of outcome events and the limited precision of the estimates. The model demonstrated modest apparent discrimination (AUC=0.68; Supplementary Figure 4 in the Appendices); however, this estimate should be interpreted with caution, as model development and performance assessment were conducted on the same dataset without external validation. Model calibration was acceptable based on the Hosmer-Lemeshow test (P>0.05). Overall, these findings should be considered exploratory and hypothesis-generating and require validation in larger external cohorts.

Subgroup analysis by glycemic control

Subgroup analyses based on HbA1c categories did not demonstrate a statistically significant association with MACE; small subgroup sizes and a high proportion of missing data limited interpretation.

## Discussion

In this single-center retrospective cohort of SPK transplant recipients, we observed a 17.5% incidence of post-transplant MACE, with cardiovascular death representing the most frequent event and arrhythmia a common secondary outcome (Table 3). This rate is higher than 7.8% reported in a large UK cohort of 1,699 SPK recipients over five years, which may reflect differences in MACE definitions, cohort composition (including recipients with type 2 diabetes), follow-up duration, or the use of an expanded composite endpoint in the present study [6]. The higher MACE incidence observed in the present study should be interpreted in the context of our expanded composite endpoint. Unlike studies using a conventional three-point MACE definition limited to cardiovascular death, nonfatal myocardial infarction, and nonfatal stroke, our definition also included heart failure hospitalization and clinically significant arrhythmia. These additional components likely increased the observed event rate and may partly explain the differences between our findings and those of prior SPK cohorts. Therefore, direct comparisons with studies using narrower MACE definitions should be made with caution. The distribution of individual MACE components was markedly uneven, with cardiovascular death accounting for the majority of events and arrhythmia comprising 7 of 14 events (50% of the composite).

Consequently, the composite outcome was largely composed of mortality and arrhythmia rather than non-fatal atherosclerotic events. Given the limited number of events, component-specific analyses were underpowered, precluding a reliable assessment of differential predictors across individual MACE components. Despite the well-established metabolic benefits of SPK transplantation, these findings underscore that cardiovascular disease remains a major source of morbidity even after successful transplantation, consistent with prior studies demonstrating persistent cardiovascular risk in this population [2,3,7]. Cardiovascular disease remains the leading cause of mortality after kidney transplantation, reflecting persistent and multifactorial risk even after restoration of renal function [18,19]. This risk appears to be concentrated within the early post-transplant period, highlighting the importance of early risk stratification and intervention [6].

A major finding of this study was the association between early kidney allograft function and post-transplant cardiovascular events after SPK transplantation. In the adjusted analysis, higher one-month eGFR was associated with lower odds of post-transplant MACE (adjusted OR, 0.61 per 10 mL/min/1.73 m² increase; 95% CI, 0.38-0.97; P=0.040) (Table 5). However, the confidence interval was wide, reflecting limited precision related to the small number of outcome events. Given the small number of outcome events, the adjusted odds ratios and P values should not be interpreted as definitive evidence of independent prediction, but rather as exploratory associations requiring validation in larger cohorts.

The direction and magnitude of this association were consistent with the univariable analysis, in which eGFR remained significantly associated with MACE (Table 4), supporting a biologically plausible relationship despite limited statistical power. These findings align with broader transplant literature demonstrating that graft dysfunction has been strongly associated with post-transplant cardiovascular events and mortality. In the FAVORIT trial, lower eGFR (<45 mL/min/1.73 m²) was independently associated with a 15% lower risk of cardiovascular disease and death per 5 mL/min/1.73 m² decrement [8]. This relationship likely reflects persistent chronic kidney disease-related pathophysiology, including inflammation, endothelial dysfunction, oxidative stress, and accelerated atherosclerosis [4,5,18].

Unlike later measures of graft function, one-month eGFR reflects early allograft performance and may represent a clinically relevant marker associated with post-transplant cardiovascular risk. However, because one-month eGFR is measured after transplantation, some MACE events (particularly arrhythmias) may have occurred before or concurrent with this measurement, and the temporal relationship cannot be definitively established. These findings support a conceptual model in which early graft function and transplant-related factors may be important markers associated with cardiovascular risk in the early post-transplant period.

The multivariable model demonstrated modest apparent discrimination (AUC=0.68). However, because model development and performance assessment were done in the same dataset without external validation, this estimate should be interpreted with caution. The wide confidence intervals further reflect limited precision. Accordingly, the model should be considered hypothesis-generating rather than predictive, and validation in larger external cohorts is needed to assess its generalizability and clinical utility.

Our findings also highlight a potential role for perioperative and donor-related factors, particularly ischemia-related injury, in shaping post-transplant cardiovascular risk [20-22]. Recipients who developed MACE had longer cold ischemic times and pancreas preservation times (Table 1). Although cold ischemic time was not significantly associated with MACE in the multivariable model (P=0.057), its consistent directionality supports a biologically plausible pathway in which ischemia-reperfusion injury impairs early graft function, thereby increasing downstream cardiovascular risk. Each additional hour of cold ischemia time has been shown to proportionally increase the risk of both graft failure and mortality in kidney transplantation [21]. Ischemia-reperfusion injury is a well-recognized driver of graft dysfunction and inflammation in transplantation, which may contribute to long-term adverse outcomes [22,23].

Notably, donor BMI was significantly higher among recipients who developed MACE (27.5±4.6 vs 21.1±11.3 kg/m², P=0.001), representing the most statistically significant univariate finding in this study. Higher donor BMI has been associated with inferior pancreas and kidney allograft outcomes in SPK transplantation, particularly at BMI ≥35 kg/m² [24-26]. Although donor BMI was not included in the multivariable model due to events-per-variable constraints, this finding warrants further investigation as a potential contributor to graft quality and downstream cardiovascular risk [16,17,24].

Taken together, these findings suggest that perioperative injury, organ preservation factors, and donor characteristics may contribute to early graft function, which in turn appears to be associated with post-transplant cardiovascular outcomes.

The counterintuitive observation that prior macrovascular comorbidities were more prevalent among recipients without MACE (48.5% vs 21.4%, P=0.080) deserves comment. This paradox may reflect selection bias inherent in transplant candidacy, whereby patients with known cardiovascular disease undergo more rigorous pre-transplant optimization, including coronary revascularization and aggressive risk factor management, potentially conferring a protective effect in the post-transplant period. Alternatively, this finding may reflect survivor bias or the small sample size and should be interpreted with caution.

At the metabolic level, baseline triglycerides were higher among recipients who developed MACE (Table 2). However, subgroup analyses based on HbA1c categories did not demonstrate a statistically significant association with MACE, and small subgroup sizes and a high proportion of missing data limited our interpretation. Although metabolic dysfunction may contribute to the overall cardiovascular risk burden, its independent role in early post-transplant outcomes appears limited in this study. These findings suggest that metabolic factors may play a contributory, but not dominant, role, with their effects potentially attenuated or overshadowed by graft function and transplant-specific factors in the early post-transplant period [2,3,5].

The TyG index, a surrogate marker of insulin resistance, was not independently associated with MACE in this cohort. This contrasts with prior studies demonstrating its association with cardiovascular risk and mortality in broader populations and in kidney transplant recipients specifically, suggesting that traditional metabolic risk markers may have limited discriminatory value in the early post-transplant period, when transplant-specific factors predominate [9-12].

Recent data suggest that longitudinal changes in the TyG index, rather than baseline values alone, may better capture dynamic metabolic risk after transplantation [9]. In a study of 106 SPK recipients with type 2 diabetes, a "metabolic worsening" TyG trajectory was associated with significantly higher MACE incidence (adjusted hazard ratio (HR) 3.52, 95% CI 1.17-10.6). In contrast, baseline TyG alone was insufficient for risk stratification [9]. The null finding for baseline TyG in the present study is consistent with this observation and supports a context-dependent role for this marker in SPK populations.

Consistent with this interpretation, post-transplant insulin dependence was more frequent among recipients who developed MACE (35.7% vs 7.6%, P=0.013 by Fisher's exact test), suggesting incomplete metabolic recovery. Of note, the univariable logistic regression yielded a non-significant association (OR 2.78, 95% CI 0.78-9.90, P=0.115), likely reflecting the different statistical properties of these tests in settings with small cell sizes. This finding highlights the heterogeneity of metabolic normalization following SPK transplantation and supports the concept that persistent insulin resistance or impaired β-cell reserve may contribute to ongoing cardiovascular risk. However, the lack of an independent association after multivariable adjustment suggests that insulin use is likely a reflection of underlying disease severity rather than a direct causal factor.

Taken together, our findings support a conceptual model in which the interplay between early graft function, perioperative injury, and residual metabolic burden shapes post-transplant cardiovascular risk in SPK recipients [4]. Within this framework, the early post-transplant period represents a critical window during which transplant-specific factors may have important associations with cardiovascular outcomes. In contrast, metabolic factors contribute in a secondary, context-dependent manner [5,6,20].

Clinical implications

These findings have several important clinical implications. First, optimizing and preserving kidney allograft function may represent a key strategy to mitigate cardiovascular risk in SPK recipients. These findings emphasize the importance of early post-transplant renal function as a marker associated with cardiovascular events after SPK transplantation. Second, minimizing ischemic injury through improved donor selection, organ preservation, and perioperative management may improve cardiovascular outcomes, potentially by preserving early graft function [20-22]. Third, although metabolic markers such as glycemic control (HbA1c) remain clinically relevant, clinical monitoring strategies may consider early post-transplant renal function as one factor associated with cardiovascular outcomes, particularly in the early post-transplant period. Fourth, the significant association between donor BMI and MACE warrants consideration in donor selection and organ allocation decisions [21].

Collectively, these findings support a clinical monitoring framework that prioritizes early graft function and highlights the early post-transplant period as a critical window for targeted intervention to reduce downstream cardiovascular risk.

Limitations

This study has several limitations. The single-center design and modest sample size limit generalizability and statistical power. Because exact cardiovascular event dates were not consistently available, MACE was analyzed as a binary yes/no outcome rather than as a time-to-event outcome. This approach precluded survival analyses and limited the temporal resolution of cardiovascular risk. Consequently, early and late events could not be distinguished, and the analysis could not account for censoring, differences in follow-up duration, or event timing. This constraint may obscure time-dependent relationships between predictors and outcomes.

The use of an expanded MACE definition, including arrhythmia and heart failure hospitalization, differs from the conventional three-point MACE composite used in many cardiovascular studies and may limit comparability with prior reports [15]. In particular, arrhythmia accounted for a substantial proportion of composite events in this cohort, which may have influenced the overall MACE incidence. Because the number of outcome events was limited, component-specific analyses were not performed.

The limited number of outcome events constrained model complexity and contributed to imprecision in effect estimates. With an events-per-variable ratio of 7, below the traditional threshold of 10, regression coefficients may be biased, and confidence intervals may have improper coverage [16,17]. In addition, the inclusion of post-transplant variables, such as one-month eGFR, reflects intermediate markers of graft function rather than baseline predictors, thereby limiting causal inference and precluding definitive conclusions regarding temporality. Residual confounding from unmeasured variables, including donor BMI, which was significantly different between groups but not included in the multivariable model, cannot be excluded.

Therefore, these findings should be interpreted as hypothesis-generating and require validation in larger prospective cohorts. Accordingly, adjusted odds ratios and P values should be interpreted cautiously and should not be considered definitive evidence of independent prediction. Additionally, the lack of external validation limits the model's generalizability and clinical applicability.

## Conclusions

In SPK transplant recipients, early post-transplant kidney allograft function was associated with post-transplant cardiovascular events. Because one-month eGFR was measured after transplantation and exact cardiovascular event dates were not consistently available, this finding should be interpreted as associative rather than causal or predictive. Given the limited number of events and potential for overfitting, these results should be interpreted cautiously and considered hypothesis-generating. Validation in larger prospective cohorts with time-to-event data is warranted.

## Figures and Tables

**Figure 4:**
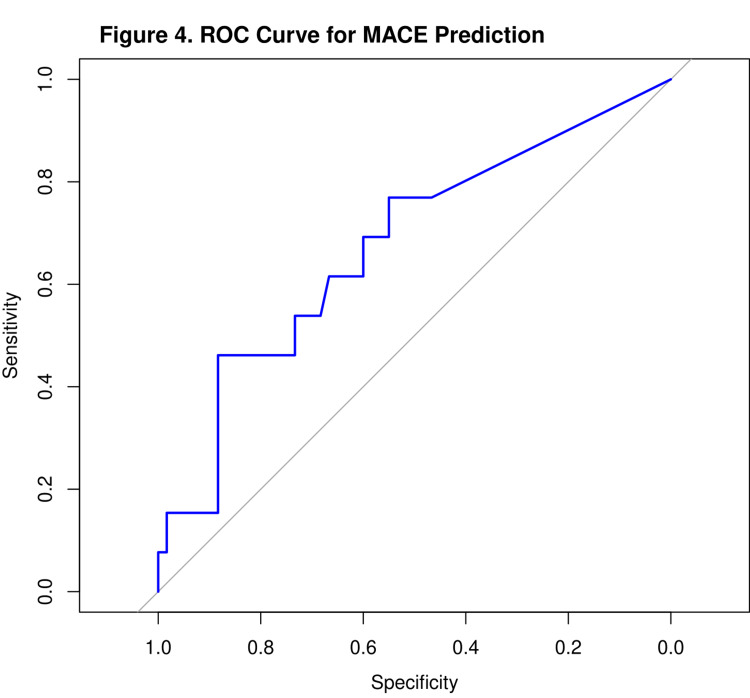
Receiver operating characteristic curve (ROC) for the multivariable model of post-transplant MACE. The ROC curve shows the apparent discrimination of the parsimonious multivariable logistic regression model, which included one-month eGFR and kidney cold ischemic time. The area under the curve was 0.68, consistent with modest discrimination. Because model performance was evaluated in the same cohort used to develop the model and the number of outcome events was limited, this estimate should be interpreted cautiously. External validation is needed before clinical prediction use.
